# Generation and Characterization of an *Nxf7* Knockout Mouse to Study *NXF5* Deficiency in a Patient with Intellectual Disability

**DOI:** 10.1371/journal.pone.0064144

**Published:** 2013-05-13

**Authors:** Lieselot Vanmarsenille, Jelle Verbeeck, Stefanie Belet, Anton J. Roebroek, Tom Van de Putte, Joke Nevelsteen, Zsuzsanna Callaerts-Vegh, Rudi D’Hooge, Peter Marynen, Guy Froyen

**Affiliations:** 1 Human Genome Laboratory, VIB Center for the Biology of Disease, Leuven, Belgium; 2 Human Genome Laboratory, Department of Human Genetics, KU Leuven, Leuven, Belgium; 3 Experimental Mouse Genetics, Department of Human Genetics, KU Leuven, Leuven, Belgium; 4 Laboratory of Molecular Biology (Celgen), Department of Human Genetics, KU Leuven, Leuven, Belgium; 5 Laboratory of Biological Psychology, KU Leuven, Leuven, Belgium; 6 Leuven Institute for Neuroscience and Disease (LIND), KU Leuven, Leuven, Belgium; The John Curtin School of Medical Research, Australia

## Abstract

Members of the Nuclear eXport Factor (NXF) family are involved in the export of mRNA from the nucleus to the cytoplasm, or hypothesized to play a role in transport of cytoplasmic mRNA. We previously reported on the loss of *NXF5* in a male patient with a syndromic form of intellectual disability. To study the functional role of NXF5 we identified the mouse counterpart. Based on synteny, mouse *Nxf2* is the ortholog of human *NXF5*. However, we provide several lines of evidence that mouse Nxf7 is the actual functional equivalent of NXF5. Both Nxf7 and NXF5 are predominantly expressed in the brain, show cytoplasmic localization, and present as granules in neuronal dendrites suggesting a role in cytoplasmic mRNA metabolism in neurons. Nxf7 was primarily detected in the pyramidal cells of the hippocampus and in layer V of the cortex. Similar to human *NXF2*, mouse *Nxf2* is highly expressed in testis and shows a nuclear localization. Interestingly, these findings point to a different evolutionary path for both *NXF* genes in human and mouse. We thus generated and validated *Nxf7* knockout mice, which were fertile and did not present any gross anatomical or morphological abnormalities. Expression profiling in the hippocampus and the cortex did not reveal significant changes between wild-type and *Nxf7* knockout mice. However, impaired spatial memory was observed in these KO mice when evaluated in the Morris water maze test. In conclusion, our findings provide strong evidence that mouse Nxf7 is the functional counterpart of human *NXF5*, which might play a critical role in mRNA metabolism in the brain.

## Introduction

Members of the Nuclear Export Factor (NXF) family play a crucial role in the export of processed mRNA from the nucleus to the cytoplasm [1]. In human, four functional *NXF* family members have been described of which three cluster on Xq22.1 [2], [3]. The ubiquitously expressed *NXF1*, which is located on chromosome 11, codes for the predominant mRNA nuclear export factor [1]. Tissue distribution of *NXF2* and *NXF3* appears to be restricted to the testis [4], whereas *NXF5* mRNA could be detected in fetal brain and hippocampus [2]. Subcellular localization revealed that NXF1 and NXF2 are predominantly nuclear, whereas the cytoplasmic presence of NXF3 and NXF5 suggests that they have a different cellular function [2], [3]. Both NXF1 and NXF2 bind mRNA, interact with NXT/p15 as well as with nucleoporins, and display RNA nuclear export activity [5]. NXF3 also has the ability to export mRNA from the nucleus to the cytoplasm but it uses an entirely different mechanism [4]. For NXF5 however, no export activity was reported although it binds non-specifically to RNA but not to nucleoporins [2].

Apart from the autosomal *Nxf1* gene the mouse also has three additional *Nxf* genes that cluster on the X chromosome (*Nxf2*, *Nxf3* and *Nxf7*). Clear orthology was established for human *NXF3* and mouse *Nxf3* [6] but the relationships of *Nxf2* and *Nxf7* to their human counterpart remained uncertain. Mouse Nxf2 localizes to the nucleus while Nxf7 is cytoplasmic [7], [8]. Furthermore, Nxf2 but not Nxf7, exports mRNA from the nucleus [8], [9]. These functional characteristics pointed towards mouse Nxf7 as the functional equivalent of human NXF5. However, while mouse *Nxf2* shows highest expression in the testis with a crucial role in spermatogenesis [10], it is also expressed in the brain as detected at mRNA [8] and protein levels [7]. *Nxf7* mRNA transcripts on the other hand, were exclusively detected in brain [2], [8]. Interestingly, Nxf7 was suggested to play a role in dendritic mRNA transport, stability or storage [9], [11]. For Nxf2 a role in mRNA dynamics in neurons was proposed based on its interaction with motor proteins [12].

We previously reported on an intellectually disabled (ID) patient who lacks NXF5 due to a pericentric inversion on the X chromosome [2]. Because of its proposed functional characteristics, deletion of NXF5 could well account for the ID observed in our patient. We identified Nxf7 as the functional mouse counterpart for human NXF5 and generated an *Nxf7* knockout (KO) mouse by a Cre-loxP-mediated out-of-frame deletion of exon 3. Based on morphological, histological, molecular and behavioural analyses of these *Nxf7* KO mice, we provide strong evidence that Nxf7 is the functional equivalent of NXF5 and that dysfunction of NXF5 could relate to cognitive impairment.

## Materials and Methods

### Nxf Genomic and mRNA Expression Analysis

Genomic analysis of the human and mouse *NXF*/*Nxf* gene clusters was performed using information from the Ensembl (http://www.ensembl.org/) and UCSC (http://genome.ucsc.edu/) web browsers. Genomic and cDNA sequences were analyzed in VectorNTi (Lifetechnologies). Primer sequences for regular and quantitative PCR (qPCR) were purchased from IDT (Integrated DNA technologies, Haasrode, Belgium) and can be found in Table S1. Human *NXF* gene expression was measured from purchased total RNA from different tissues (Lifetechnologies) while for mouse *Nxf* genes, total RNA was extracted from tissues of C57Bl/6 mice with Trizol (Lifetechnologies) and treated with DNaseI. cDNA synthesis and qPCR was performed as described earlier [13]. For regular PCR, primers were chosen within or flanking exon 3 to discriminate between wild type (WT) and KO loci. qPCR was performed as described earlier [13] using SYBRgreen on a LC480 instrument (Roche). Relative quantitation by qPCR of *NXF*/*Nxf* expression (exon 10–11 for *NXF2*; exon 15 for *NXF5;* exon 21–22 for *Nxf2;* exon 3–4 and exon 21–22 for *Nxf7*) was normalized towards the housekeeping genes *GUSB/Gusb* and *HPRT1/Hprt1*. Genomic DNA contamination was excluded using a non-reverse-transcribed RNA sample. Equal amplification efficiencies of qPCR primer sets for *Nxf2* and *Nxf7* were demonstrated by qPCR on equal amounts of plasmid constructs (data not shown). qPCR primers were chosen in regions present in all known Nxf transcripts.

The different mouse *Nxf* genes were cloned from cDNA obtained from testis (*Nxf2* and *Nxf3*) and brain (*Nxf1* and *Nxf7*) tissues and cloned into the pBluescript-KS (Stratagene) or pEGFP-C1 (Clontech) vectors.

### Generation of Nxf7 KO Mouse and Genotyping

We selected the *Nxf7* exon 3 for targeted deletion because of the minimum amount of sequence similarity with the other *Nxf* genes, it is included in both reported *Nxf7* isoforms, and is predicted to result in an out-of-frame transcript. A genomic mouse *Nxf7* gene-containing clone of 129 Sv genetic background was obtained by screening a BAC library with a 429 bp *Nxf7*-specific probe. From the single positive BAC clone (449B22), a 6.5 kb BamHI fragment containing exon 1 to exon 5 was cloned in the pUC18 vector. From this construct, a 2.3 kb SalI/BamHI fragment harbouring exons 3 to 5 was subcloned into pUC18. Subsequently, a loxP-frt-*PGK* promoter-Hygromycin B (Hyg)-frt cassette was cloned into the EcoRV site of intron 2, and a loxP site was inserted into a blunted BglII site within intron 3 of the *Nxf7* gene. In this way, exon 3 was flanked by two loxP sites with a Hyg selection marker in between both loxP sites (Fig. S1). Finally, a 4.2 kb BamHI/SalI fragment which harbours exons 1 and 2 was re-ligated using the SalI site in intron 2. This final construct carries 5 kb Nxf7 genomic sequences upstream of the proximal loxP site and 1 kb downstream of the distal loxP site. 129 Sv ES cells were electroporated with the construct and after Hyg selection cells were cloned by limiting dilution and grown in 96-well plates. ES clones were grown in duplicated plates and DNA was extracted from one set. Positive clones resulting from homologous recombination were identified by PCR using a forward primer within the distal loxP site and the reverse primer in exon 6, not included in the construct. From the 196 clones screened, two (clones 54 and 175) were positive (1% efficiency). Southern blot with a 429 bp *Nxf7* 5′-probe on PstI-digested genomic DNA of both positive clones demonstrated correct targeted recombination of the fragment without any additional unexpected bands (Fig. S1). Next, clone 54 was injected in blastocysts of a C57Bl/6 mouse as described elsewhere [14]. Chimeric offspring were checked by PCR as described above and positive males were used for further breeding with C57Bl/6 female mice. Subsequent, to successful germline transmission these mice were crossed with female C57Bl/6 mice expressing Cre recombinase from the ubiquitous PGK promoter. For genotyping, DNA from tail fibroblasts was isolated and PCR was performed with three primer pairs resulting in reliable discrimination (Fig. S2). Primer sequences can be found in Table S1. Mice were bred into the C57Bl/6 background for at least 10 generations. Standard breeding was done by crossing heterozygous (HTZ) *Nxf7* females with C57Bl/6 WT males. In this breeding scheme we observed the following numbers of offspring per genotype out of a total of 259 pups: 45 KO males (17%), 78 WT males (30%), 57 HTZ females (22%) and 79 WT females (31%), which is significantly different from the predicted Mendelian segregation (p = 0.02). The successful mating of male KO mice with C57Bl/6 WT females and female KO mice with C57Bl/6 WT males demonstrated that these animals are fertile.

### Immunohistology, Immunofluorescence and In Situ hybridization

Six week old WT and KO male mice were anesthetized and transcardially perfused with 4% paraformaldehyde in PBS. Immunostaining of paraffin-embedded brain slices was done with hematoxylin and eosin (HE; Merck). Staining of neurons was performed with anti-NeuN antibody (1/4000; Chemicon) followed by biotinylated rabbit anti-mouse antibody (1/400; Sigma), and peroxidase-labeled streptavidine (1/100; Perkin Elmer) according to standard procedures. Stained sections were analyzed with an Axioplan2 fluorescent microscope (Carl Zeiss, Göttingen, Germany) and inspected for morphological alterations in the brains of KO mice. Immunofluorescence for Nxf7 was performed on 6 µm thick paraffin brain sections from WT and *Nxf7* KO mice. Polyclonal anti-Nxf7-Nt Ab (1∶200; see below) was incubated overnight. As a secondary antibody we used an Alexa Fluor 568 goat anti-rabbit IgG. Whole mount and tissue slice in situ hybridizations were performed as described previously [15] using DIG-UTP-labeled *Nxf2*- and *Nxf7*-specific RNA riboprobes and visualized with an anti-DIG-Cy3 Ab or with NBT/BCIP as described elsewhere [16].

### Microarray Expression Analysis

For microarray expression analysis, hippocampi and cortices were dissected from 6-week old WT and *Nxf7* KO male mice. RNA concentration and integrity was assessed using a Bioanalyzer 2100 (Agilent). Per sample, an amount of 1 µg of total RNA spiked with 10 viral polyA transcript controls (Agilent) was converted to double stranded cDNA in a reverse transcription reaction. Subsequently, the samples were converted to antisense cRNA, amplified and labeled with Cyanine 3-CTP (Cy3) or Cyanine 5-CTP (Cy5) in an in vitro transcription reaction according to the manufacturer’s protocol (Agilent). Cy5- and Cy3-labeled samples were co-hybridised in a loop-design fashion (WT1/KO1, KO1/WT2, WT2/KO2 and KO2/WT1) on Agilent Mouse Whole Genome arrays. Arrays were scanned with the Agilent DNA MicroArray Scanner with Surescan High-Resolution Technology and probe signals were quantified using Agilent’s Feature Extraction software (version 10.1.1.1). Statistical data analysis was performed on the processed Cy3 and Cy5 intensities, as provided by the Feature Extraction Software version 10.1. Probes with none of the eight signals flagged as positive and significant (by the Feature Extraction Software) were omitted from all subsequent analyses as well as the various controls. R was used in combination with the limma package to perform the microarray data analysis [17], [18]. Subsequently, the contrast between the KO and WT samples was extracted. Testing whether a contrast is significantly different from 0 was done by using a moderated t-statistic, as implemented in Limma. Probes with a p-value smaller than 0.001 and an absolute fold-change larger than 2, were selected as differentially expressed. The data can be found in the Gene Expression Omnibus (GEO) database under accession number GSE29541.

### Western Blot

Since no commercial antibodies (Abs) against Nxf7 were available, we generated polyclonal antibodies in rabbits using standard immunization protocols. We produced Nxf7-specific Abs against the two domains that differ most from the other Nxf proteins; the N-terminal amino acids 1–91 (Nxf7-Nt) and the C-terminal 368–619 fragment (Nxf7-Ct). Rabbits were injected three times and the specificity of the final antiserum was tested on lysates of HEK293T cells transfected with flag-tagged *Nxf1*, *Nxf2*, *Nxf3* or *Nxf7* constructs cloned into pcDNA3.1 (Lifetechnologies). Anti-flag Ab (Sigma) was used to check the transfection efficiency. For the anti-Nxf7 Abs, peroxidase-conjugated goat anti-rabbit Ig was used as the second Ab. Signals were visualized with the LAS-3000 Imaging System (Fujifilm Global). Cortical and hippocampal protein lysates (30 µg) were analyzed according to standard procedures. Protein lysates were also prepared from dissected yolk sacs from embryos of a pregnant *Nxf7* HTZ female, mated with an *Nxf7* KO male. Genotyping was performed on DNA extracted from the tails of the embryos as described earlier. Western blot was performed on 30 µg of protein lysates separated on a 4–12% NUPAGE bis-Tris gel (Lifetechnologies). The Nxf7-Ct Ab was used as the primary Ab.

### Behavioural Assays

To assess neuromotor abilities, motor coordination on the accelerating rotarod and grip strength were recorded as described earlier [19].

Male *Nxf7*-deficient (*Nxf7* KO, n = 21) mice and their wild-type (WT, n = 20) littermates (age 2–3 months) were compared in a hippocampus-dependent spatial learning test [19]. Animals were kept in standard animal cages under conventional laboratory conditions (12 h/12 h light-dark cycle, 22°C), with ad libitum access to food and water. Experiments were conducted during the light phase of the activity cycle. Hippocampus-dependent spatial memory abilities were examined in the standard hidden-platform acquisition and retention (i.e., long-term memory) versions of the Morris maze [20]. A 150 cm circular pool was filled with water, opacified with non-toxic white paint, and kept at 26°C as previously described [19]. A 15 cm round platform was hidden 1 cm beneath the surface of the water at a fixed position. Each daily trial block consisted of 4 swimming trials (10 min inter-trial interval) starting randomly from each of 4 starting positions. Mice that failed to find the platform within 2 min were guided to the platform, where they remained for 15 s before being returned to their cages. To test spatial memory, probe trials (100 s) by removing the platform were conducted at the end of a working week. Time spent in target quadrant compared to chance level (25%) was used as measure for long-term retention memory. Swimming paths of the animals were recorded using EthoVision video tracking equipment and software (Noldus bv, Wageningen, The Netherlands). Statistical analysis was performed using SigmaStat for Windows 3.11 or GraphPad Prism 5.0. Data are presented as means ± SEMs. Differences between groups were statistically analyzed by Student’s t-test, one sample t-test, or two-way repeated measures ANOVA when appropriate, with the power of alpha set at 0.05. For post hoc comparison, multivariate analysis with Holmes-Sidak corrections was performed.

## Results

### Mouse *Nxf7* is the Ortholog of Human *NXF5*

**Figure 1:**
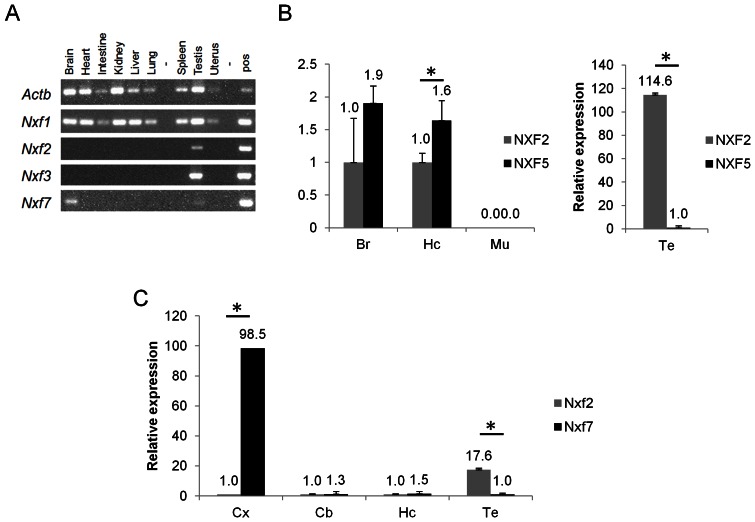
Relative expression analysis of *NXF/Nxf* in tissues. (A) RT-PCR for mRNA level quantification of *Nxf1*, *Nxf2*, *Nxf3* and *Nxf7* in mouse tissues. *ActB* was used as the housekeeping gene. (B) RT-qPCR for relative expression analysis of human *NXF5* and *NXF2* normalized to *GUSB* and *HPRT1*. (C) RT-qPCR for relative expression of mouse *Nxf2* and *Nxf7*, normalized to *Gusb* and *Hprt1*. Cx, cortex; Hc, hippocampus; Cb, cerebellum; Te, testis; Br, Brain; Mu, muscle. N = 3. Means ± standard deviations. *p<0.05.

The three functional human *NXF* genes cluster in a 1.27 Mb interval (101.08 to 102.35 Mb) in the orientation Xcen-*NXF5-NXF2-NXF3*-Xqter (UCSC build hg19, Feb 2009). The mouse syntenic region covers 1.22 Mb (131.46 to 132.68 Mb) (UCSC build mm9, July 2007) in the orientation Xcen-*Nxf2-Nxf7-Nxf3*-Xqter. Therefore, the syntenic regions including the additional genes located in between the *NXF/Nxf* genes, strongly indicate that the human *NXF5*, *NXF2* and *NXF3* genes are the orthologs of mouse *Nxf2*, *Nxf7* and *Nxf3*, respectively (illustrated in Fig. S3). Indeed, the UCSC genome browser points to *NXF5* as the ortholog for *Nxf2*, while *NXF2* is indicated to be the ortholog for *Nxf7*. However, based on literature and protein alignments (Fig. S4) their functional relations remained unclear. First, we evaluated theexpression of mouse *Nxf* genes in different tissues, since contradictory results have been reported in the literature. By regular PCR, mouse *Nxf2* and *Nxf3* showed selective expression in the testis, while *Nxf7* mRNA was predominately detected in adult mouse brain and at lower levels in testis (Fig. 1A). Furthermore, we relatively quantified human *NXF2* and *NXF5* mRNA levels in hippocampus, whole brain, muscle and testis. *NXF5* transcript levels in human hippocampus and whole brain were about 2-fold higher than those of *NXF2* (Fig. 1B). No expression was detected in the muscle as well as other tissues tested (data not shown. In the testis, *NXF2* mRNA levels were about 100-fold higher than those of *NXF5*. We evaluated the same brain regions in the mouse, revealing a 100-fold higher *Nxf7* expression in the cortex in comparison to *Nxf2* (Fig. 1C). In the hippocampus, the *Nxf7* levels were 32-fold lower than found in cortex and only 1.5-fold higher than *Nxf2* levels. However, in the testis *Nxf2* mRNA was >15-fold more abundant compared to *Nxf7*. In the cerebellum, transcript levels were equally low for both *Nxf* genes (**Fig.** 1C). Moreover, expression levels of both genes remained stable in cortex and hippocampus at least during the first 12 weeks of life (data not shown).

**Figure 2:**
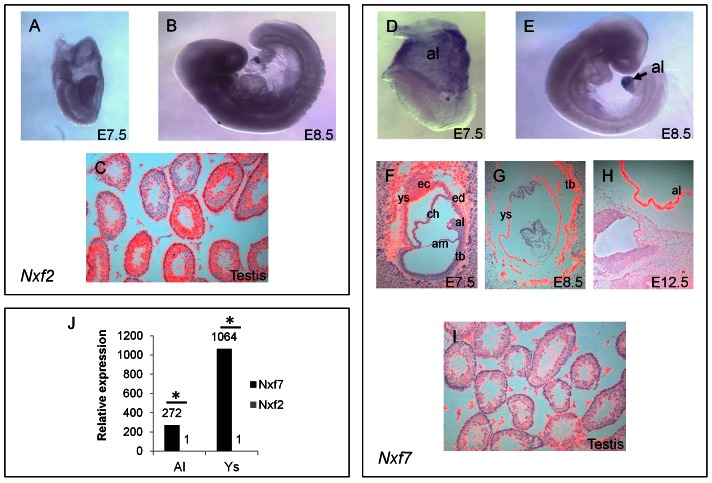
In situ hybridization of mouse *Nxf2* and *Nxf7*. Lateral view of E7.5 and E8.5 embryos and sections of E12.5 testis, hybridized with the *Nxf2* (A,B,C) or *Nxf7* (D-I) antisense probes. *Nxf7* mRNA is highly expressed in the allantois (D,E,F,H), chorion, yolk sac and giant cells of the trophoblast (F,G). al, allantois; ys, yolk sac; tb, trophoblast; ch, chorion; am, amnion; ec, ectoplacental cone; ed, extra-embryonal endoderm. No expression for *Nxf2* could be detected in these tissues at any time point. *Nxf2* is abundantly expressed in the adult testis (C) while much less staining was obtained for *Nxf7* (I) in this tissue. (J) RT-qPCR for relative expression of mouse *Nxf2* and *Nxf7*, normalized to *Gusb* and *Hprt1*. Al, allantois; Ys, yolk sac; N = 2. Means ± standard deviations. *p<0.05.

**Figure 3:**
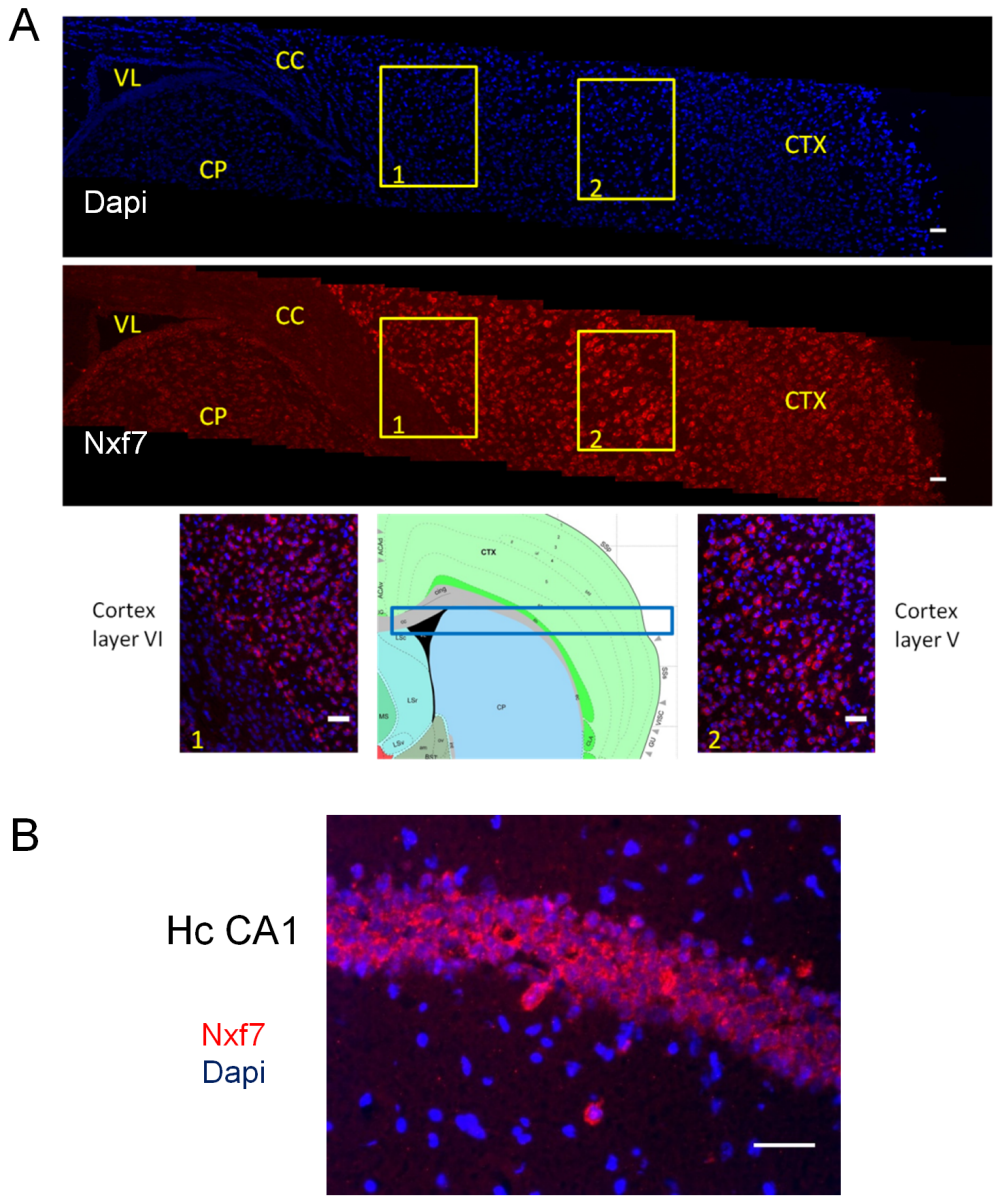
Expression of mouse *Nxf7* in adult mouse brain as detected by in situ hybridization. (A) Cortical *Nxf7* signals (upper panel) and Dapi staining (middle panel). The lower panels show the *Nxf7*/Dapi-merged pictures of the yellow boxed region 1 (border between CC and CTX) and region 2 (layers V and VI). A schematic view of the brain is given in between both merged pictures with the brain region shown in the upper parts, indicated with a blue box. VL, lateral ventricle; CC, corpus callosum; CTX, Cerebral cortex; CP, Caudoputamen. (B) Merged *Nxf7* (red) and Dapi (blue) staining of the pyramidal cell layer of the CA1 region of the hippocampus (Hc). Experiments were done in duplicate. Scale bar, 100 µm.

To assess the mRNA localization of *Nxf2* and *Nxf7* more specifically, we performed *in situ* hybridization (ISH) on whole mouse embryos and embryonic slices (E7.5–E12.5) as well as on sections of adult testis and brain using *Nxf2-* and *Nxf7-*specific probes. ISH with the *Nxf2* antisense probe did not reveal any staining in the embryo (Fig. 2A,B). However, strong signals were noticed in the adult testes, more particular in the spermatogonia-forming inner cell layer of the tubuli seminiferi that contain the mature sperm cells (Fig. 2C). ISH with the *Nxf7* antisense probe did not reveal any staining in the E7.5 to E12.5 embryos, but intense and specific staining was observed in extra-embryonic tissues like allantois, yolk sac and membranes of amnion and chorion (Fig. 2D–H). qPCR on the allantois and yolk sac confirmed the high *Nxf7* levels while *Nxf2* was barely detected in these tissues (Fig. 2J) validating our ISH data. In the testis, some *Nxf7* staining was noticed in the interstitial cells although signals were low (Fig. 2I). Finally, ISH with the *Nxf7* antisense (Fig. 3) but not the sense probe (Fig. S5) revealed a more intense staining in layer V of the cortex, and the pyramidal cells of the CA1 region of the hippocampus. Taken together, the ISH data confirm our qPCR data showing highest expression of *Nxf7* in extra-embryonic tissues during early embryogenesis and moderate levels in the brain.

Overexpression of *GFP-Nxf* fusion genes in human 293T cells was performed to check the subcellular localization of the mouse Nxf proteins. Mouse Nxf1 and Nxf2 clearly localize in the nucleus while Nxf7 stains the cytoplasm of these cells (Fig. S6). Treatment of the cells with Triton X-100 revealed staining of the nuclear rim for Nxf1 and Nxf2 but not for Nxf7, which demonstrated that Nxf7 does not bind to the nucleoporins of the nuclear membrane. The subcellular localization pattern of Nxf7 is thus comparable with that of NXF5 as previously reported [2]. Combining these findings with our expression data we have strong evidence to conclude that mouse Nxf7 is the functional equivalent of human NXF5.

### Generation and Validation of *Nxf7*-deficient Mice

**Figure 4:**
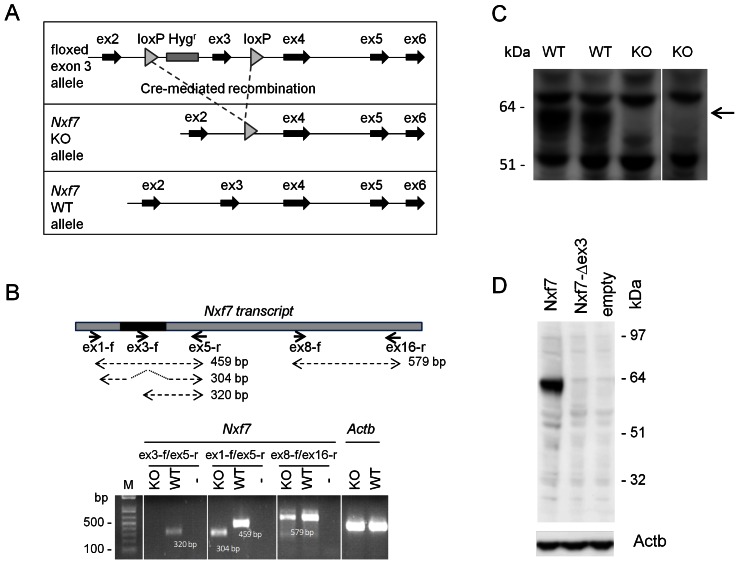
Generation and genotyping of *Nxf7* knockout mice. (A) Schematic representation of the targeted *Nxf7* allele (upper part) and its Cre-mediated KO allele (middle part). (B) Schematic representation of the *Nxf7* transcript with the PCR primers. Position of exon 3 is indicated in dark gray. Amplicon sizes are indicated in base pair (bp). The agarose gel shows the PCR data of the expression analyses for the indicated primer pairs performed on brain cDNA of KO and WT male mice. (C) Western blot on yolk sac from WT and *Nxf7* KO male embryos with the polyclonal Nxf7-Nt Ab. (D) Western blot on protein lysates obtained from transfected HEK-293T cells with the wild type *Nxf7-flag* cDNA (lane 1) and the exon3-lacking *Nxf7-flag* cDNA (lane 2). Detection was performed with anti-flag Ab. The anti-Actb Ab was used as the loading control. Western blot for the HA-tag gave the same result (data not shown).

**Figure 5:**
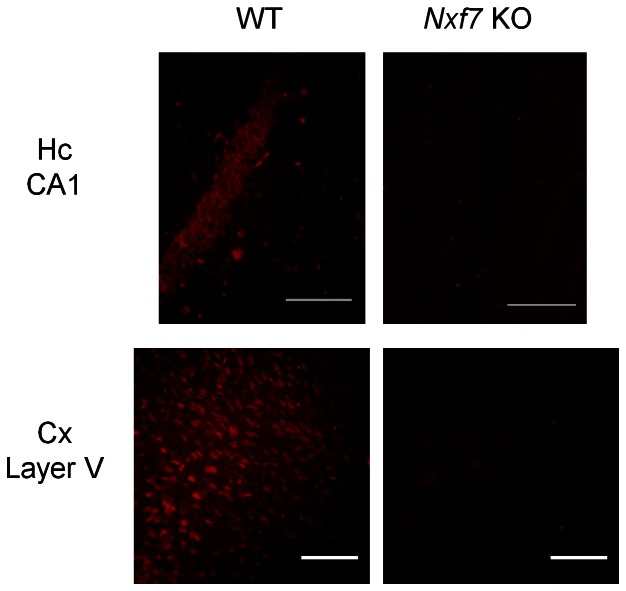
Immunofluorescence of Nxf7 on brain sections. Staining with the polyclonal anti-Nxf7-Nt antibodies on hippocampus (Hc) CA1 (upper panels) and cortex (Cx) layer V (lower panels) sections of wild type (WT) and *Nxf7* knockout (KO) mice. The assay was done in duplicate. Images were taken with a Leico microscope at 20×magnification. Scale bar, 100 µm.

*Nxf7* KO mice were produced via targeted deletion of a floxed *Nxf7* exon 3 (illustrated in Fig. 4A). Deletion of exon 3 was achieved through mating of a knock-in male mouse with female PGK-Cre-expressing mice. For *Nxf7* expression analysis in the brain and testis, a first primer pair in exon 1 and 5 yielded a product of 459 bp and 304 bp for the WT and KO alleles, respectively (Fig. 4B). The second primer set in exon 3 and 5 resulted in a product of 320 bp for the WT allele only, thus demonstrating the absence of exon 3 in the KO mouse. Finally, primers in exon 8 and 16 provided a PCR band of 579 bp for both alleles indicating, as expected, that an exon 3-lacking *Nxf7* transcript was produced in the KO mouse irrespective of the premature stop codon in exon 4. qPCR on the *Nxf7* KO samples validated the regular PCR data since the *Nxf7* primer pair in exon3–4 did not yield any product while the primer pair in exon21–22 showed mRNA abundances that were about half of those detected in the corresponding WT samples (data not shown). At the protein level, we were unable to detect Nxf7 in WT brain samples using our polyclonal antibodies. However, when using WT yolk sac lysate, which has higher levels of *Nxf7* mRNA, we could visualize an Nxf7 band at about 65 kDa while no band was observed for the KO samples (Fig. 4C). Moreover, we did not detect any additional lower band from a potential smaller Nxf7 protein fragment. To provide additional evidence that no alternative protein was produced in the KO mice, we generated an exon 3-lacking *Nxf7* construct flanked by a Flag tag at the N-terminal side and an HA tag at its C-terminus. Upon transfection in HEK-293T cells, we did not observe a smaller protein fragment by Western blot using antibodies against both tags (Fig. 4D). Furthermore, immunofluorescent staining on brain slices of WT and *Nxf7* KO mice, revealed a specific staining in the CA1 pyramidal cells of the hippocampus as well as layer V of the cortex in WT but not in *Nxf7* KO brains (Fig. 5), again demonstrating the lack of Nxf7 protein in our *Nxf7* KO mouse.

### Morphological and Molecular Analysis of Nxf7 KO Mice

Because of the expression of Nxf7 in the brain, we investigated the gross morphology of this tissue using HA and NeuN staining on coronal brain slices of WT and KO mice but we did not find any apparent histological changes (data not shown). Next, expression profiling by microarray analysis of hippocampi and cortices of both genotypes was performed. Data were analyzed for each individual hybridization experiment and log_2_ ratios were calculated for each gene. Pairwise comparisons of the ratios for the different hybridizations were done in order to search for highly similar ratios between WT1/KO1 and WT2/KO2 on the one hand, and KO1/WT2 and KO2/WT1 on the other hand. For the hippocampus, ranking of those ratios with stable but opposite values of WT/KO and KO/WT revealed 19 up-regulated and 4 down-regulated genes with a significant >2-fold change in expression (p<0.001) between WT and KO (Fig. S7A). Verification of the data was performed for 17 selected genes using qPCR on the original as well as four additional WT and KO samples. From those, 10 genes showed high variability (>5-fold) of mRNA levels between animals of the same genotype making 2- to 3-fold expression differences between WT and KO animals irrelevant. The remaining 7 genes showed differences <1.5-fold excluding them as significantly different. For the cortex samples we found 16 genes increased >2-fold (p<0.001) in KO samples (Fig. S7B), but again after qPCR validation no consistently altered mRNA levels were found between both genotypes. Moreover, evaluation of the differentially expressed genes in hippocampus and cortex using Gorilla or Ingenuity Pathway analyses did not reveal any enriched pathways (data not shown). The data for both sets of hybridizations can be found in the Gene Expression Omnibus (GEO) database under accession number GSE29541.

### Behavioural Analysis in the Morris Water Maze

**Figure 6:**
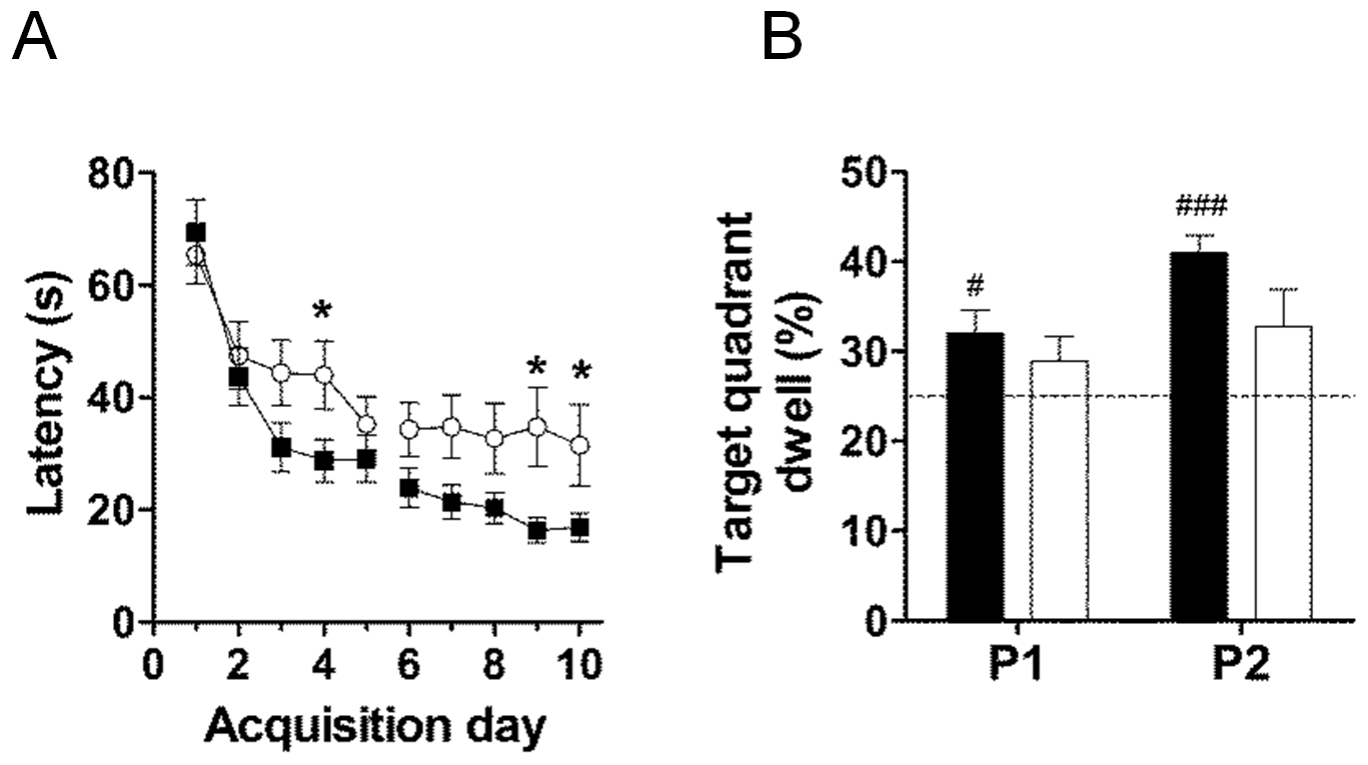
Hippocampus-dependent spatial learning in the Morris water maze. (A) *Nxf7* KO animals (n = 21, open symbols) needed significantly longer to find the hidden platform than WT animals (n = 20, black symbols) during the 10 acquisition days. (B) During probe trials, only WT animals spent significant more time in target quadrant compared to chance (dotted line at 25%), while *Nxf7* KO did not. Data are presented as means ± SEM. *p<0.05 compared to WT (Student’s t-test), # p<0.05 and ### p<0.001 compared to chance level (one sample t-test).

We hypothesized that loss of Nxf7 leads to impairment in learning and memory as has been shown in patients with a loss of NXF5. To test this prediction we analyzed the performance of the Nxf7 mouse in the Morris water maze assay. No differences were observed in the rotarod or grip strength assays indicating that gross (neuro)motor functions were not affected in the *Nxf7* KO mice. However, spatial learning in the Morris water maze was significantly impaired in the KO compared to WT mice (Fig. 6A). While there was an overall decrease in latency to find the hidden platform over acquisition days (RM ANOVA effect for factor *trial* F_9,409_ = 23.44, P<0.001), WT mice were more efficient to do so (effect for factor *genotype* F_1,409_ = 4.57, P<0.05). This impairment was not caused by differences in path length or in swim speed (data not shown). When tested for spatial retention memory, WT mice displayed significant target preference during first (P1) and second (P2) probe trial. In contrast, time spent in target quadrant was not significantly different from the *Nxf7* KO mice (Fig. 6B). Both overall path length or swim speed were not different between the genotypes during probe trials (data not shown).

## Discussion

We previously reported on a male patient with a syndromic form of ID in whom a pericentric inversion disrupted the *NXF5* gene at Xq22 [2], [21]. This patient, who thus lacks the *NXF5* gene, shows several prominent clinical characteristics that include severe ID, muscular wasting, anxiety with eye contact avoidance, and poor adaptive functioning [21]. Recently, a female ID patient was described with a 1.1 Mb deletion that also harboured the *NXF5* gene, amongst 11 others including *NXF2* [22]. Although her phenotype is worse compared to that of the patient lacking only *NXF5*, common features include severe ID and muscle hypotonia. Moreover, we reported on a patient with moderate ID carrying a 0.8 Mb duplication that harboured 15 genes including *NXF5* but no other *NXF* genes [23]. It has been reported that not only decreased but also increased gene dosages of known XLID genes can result in ID [13], [24], [25]. Consequently, *NXF5* represents a strong candidate ID gene. To test this hypothesis, we aimed to evaluate the role of NXF5 in a mouse model, however first needed to pinpoint the murine functional equivalent.

The three protein coding members of the *NXF* family that locate on the human as well as the murine X chromosome are embedded in a syntenic region with a high conservation of gene content. Based on this organization, human *NXF5* appears to be the ortholog of mouse *Nxf2,* human *NXF2* of murine *Nxf7*, and *NXF3* of *Nxf3*. This synteny suggests that the three *NXF*/*Nxf* genes on the X chromosome might have evolved before the divergence of murine and human phylogenetic lines. Notably, two additional *NXF* genes are located in the human *NXF* cluster on Xq; the *NXF2B* gene, which arose from a very recent inverted tandem duplication of *NXF2*, and the apparent pseudogene *NXF4*, which lacks an open reading frame. In the mouse genome, all three *Nxf* genes are in the indirect orientation, while in men, an indirect orientation is present for the *NXF5, NXF2B* and *NXF3* genes, indicating that *NXF2B* actually is the ancestral *NXF2* copy. The genomic exon-intron structure of human *NXF5* and *NXF3* are very similar to that of mouse *Nxf2*, *Nxf7* and *Nxf3*. That of *NXF2 (B)* differs substantially from this common organization. However, when the proposed characteristics of the X-linked NXF/Nxf proteins are taken into account, their functional relation seems to differ from their ancestral synteny. Previous studies established that *NXF2*/*Nxf2* show highest mRNA expression in testis and their protein products are predominantly localized in the nucleus. NXF5/Nxf7 proteins on the other hand, display an extensive cytoplasmic staining with a granular pattern in neurons [2]–[4], [8], [11]. We demonstrated by ISH and qPCR, high *Nxf7* mRNA levels in extra-embryonic tissues for which its function in these gestational structures remains unknown. In the brain, significant expression levels of *Nxf7* mRNA was detected in the pyramidal cell layer of the hippocampus and layer V of the cortex, which was confirmed at the protein level. Although Tan and colleagues detected highest Nxf2 levels in brain tissue by Western blot [7], our qPCR data corroborated the occurrence of only minor amounts of *Nxf2* mRNA in brain compared to approximately 250-fold higher levels in testis. Notably, cortical *Nxf7* mRNA expression was about 100-fold higher compared to *Nxf2*.

As previously reported, the subcellular localization data obtained from transfected *EGFP-NXF/Nxf* fusion constructs clearly demonstrated a nuclear localization for Nxf2 [7], [8] while Nxf7 is predominantly cytoplasmic [7]–[9], [11], resembling the localization of human NXF2 [3] and NXF5 [2], respectively. In addition, Nxf2 and NXF2 are present at the nuclear rim, which is not the case for Nxf7 and NXF5 [3]. The proposed functional relation of human NXF5 and mouse Nxf7 is strengthened by the fact that NXF2 and Nxf2 promote mRNA export, whereas NXF5 and Nxf7 do not [2], [3], [7], [8]. Taken together, our data combined with those obtained by others strongly argue in favour of mouse Nxf7 being the functional counterpart of human NXF5, which infers a divergent functional evolution of the *NXF/Nxf* genes in men and mice.

The *Nxf7* knockout mouse was generated by targeted deletion of exon 3. RT-PCR and qPCR on cDNA from male KO mice demonstrated the absence of any exon 3-containing transcript. However, this mutant transcript is still produced, although at lower levels compared to full length *Nxf7* mRNA in WT mice. Importantly, exon 3 deletion generates an out-of-frame transcript with a stop codon in exon 4 that precludes formation of a functional protein. These findings point to nonsense-mediated mRNA decay of the mutant transcript in the KO mice. Our inability to detect any Nxf7 protein in the WT cortex by Western blot likely is caused by the modest Nxf7 expression in this tissue and/or due to the low sensitivity of our polyclonal antibodies. Therefore, we used the yolk sac of embryos to demonstrate the absence of Nxf7 in the KO samples. Analysis for a potential Nxf7 protein fragment in the KO mouse, which could have been generated through the use of an alternative start codon in exon 4, was not detected. However, we realize that such a band could have been masked by background signals. Alternatively, transfection of HEK-293T cells with the exon 3-lacking construct did not reveal an N- or C-terminal Nxf7 fragment. Notwithstanding, we cannot firmly exclude the production of an Nxf7 fragment.

Strikingly, microarray analyses did not reveal specific altered transcript levels in the cortex or hippocampus of *Nxf7* KO mice compared to WT littermates using a threshold of 2.0 for differential expression. Our data thus seem to demonstrate that the changes in transcript levels are more subtle, or that the transcript profiles in these brain structures do not change upon removal of Nxf7. If so, it could be explained by the fact that Nxf7 plays a role in mRNA transport and/or storage but not in expression, translation or stability. However, analysis of more samples or analysis of the synaptoneurosomes is necessary to further investigate our proposed hypothesis.

Finally, we demonstrated an impairment in spatial learning and memory performance in the Morris water maze test, suggesting impairment in hippocampal functions in the *Nxf7* KO mice. This memory deficit could mimic the cognitive defects in *NXF5*-deficient patients.

In conclusion, we provide strong arguments to state that mouse Nxf7 is the functional equivalent of human NXF5 even though mouse Nxf2 is syntenic with NXF5, suggesting a differential evolutionary path of members of this gene cluster in both species. We then generated an *Nxf7* knockout mouse as an animal model to study *NXF5* loss-of-function mutations in patients. Initial behavioural characterization of these mice revealed significantly reduced spatial learning. Given the exclusive localization of Nxf7 in cytoplasmic granules including translating ribosomes, stress granules and P-bodies [9], [11], its association with hnRNP A3 [11] and its co-localization with the neuronal mRNA transport marker Stau1 [9], a role for Nxf7 is suggested in mRNA cytoplasmic transport, translation, degradation and/or storage. Therefore, next to FMRP for which its absence results in Fragile-X syndrome [26], NXF5 seems to be a second ID-related protein involved in mRNA metabolism in neurons. Identification of associated mRNAs, evaluation of additional differences in behaviour as well as electrophysiological studies in the KO mice might shed light on its presumed role in the brain and establish a potential role in ID or other clinical features observed in our patient.

## Supporting Information

Figure S1 **Confirmation of correctly targeted recombination in clones 54 and 175.** (A) schematic representation of the transgenic *Nxf7*-targeted allele and the WT allele with the localization of the probes used in Southern blot. B, BamHI; P, PstI site; Hyg, hygromycine-resistant marker. Position of the probe in *Nxf7* intron 2 is given as a horizontal blue bar; positions of primers a and b as black arrows. (B) PCR on genomic DNA of transfected ES clones with primer a, binding in the loxP sequence, and primer b annealing in exon 6 just outside the transfected construct. The targeting plasmid used for transfection was the positive control. This plasmid harbours the 8.7 kb BamHI (indicated by ‘B’) fragment shown on the construct of the ‘Transgenic *Nxf7* allele’. (C) Southern blot analysis on PstI-digested DNA of embryonic stem (ES) WT cells and ES clones 54, 127 and 175 with the ^32^P-labeled probe.
(TIF)

Figure S2 **Genotyping of the** ***Nxf7*** **KO mice with three primer sets.** (A) Schematic representation of the targeted *Nxf7* allele (upper part) and its Cre-mediated KO allele (middle part). PCR primers (arrows) for genotyping are indicated with their respective amplicon sizes, and compared to the WT allele (lower part). (B) Scheme of PCR analyses to genotype the mice. The result obtained after standard agarose gel electrophoresis is provided on the right.
(TIF)

Figure S3 **Schematic view of the syntenic regions in man (Xq22.1) and mouse (XqE3-F1).** The orthology of genes within this highly conserved region is illustrated. According to the immediate flanking and neighbouring genes (*GLA, ARMCX, ZMAT1, TCEAL6, GPRASP1, BEX1, BEX4*), mouse *Nxf2* is syntenic with human *NXF5,* and mouse *Nxf7* with human *NXF2*. However, based on highest expression and subcellular localization data (indicated in between brackets) as well as other functional characteristics mentioned in the manuscript, the functional equivalent of Nxf2 should be NXF2, and that of NXF5 should be Nxf7. Note that in human, two additional *NXF* genes are present: *NXF2B* and the pseudogene *NXF4*. Positions (in Mb) on the X chromosome are given at the left and right.
(TIF)

Figure S4 **Protein alignment of NXF5 with Nxf2 or Nxf7.** The human NXF5 protein sequence was aligned with that of mouse Nxf2 (A) or Nxf7 (B) using CLC DNA workbench software. Identical residues are highlighted in red.
(TIF)

Figure S5 **In situ hybridisation with the Nxf7 sense probe on brain slices revealed no specific staining.** Representative pictures are shown for the CA1 region of the hippocampus and layer V of the cortex. The assay was done in duplicate. Scale bar, 100 µm.
(TIF)

Figure S6 **Subcellular localization of mouse Nxf proteins in HEK293T cells.** The open reading frame of Nxf1, Nxf2 and Nxf7 were cloned in the pEGFP vector and 0.5 µg plasmid was transfected with the FuGENE6 transfection reagent. Cells were incubated for 20 hr and fixed with 4% formaldehyde for 15 min. Fluorescent signals were visualised with a MRC1024 confocal microscope. –Tx: without Triton-X100 treatment;+Tx; with Triton-X100 treatment. The magnifications used here slightly differ between images to accommodate the most optimal visualization.
(TIF)

Figure S7 **Microarray expression analysis of hippocampal and cortical RNA samples from male WT and Nxf7 KO littermates.** Combined analysed data are shown for the hybridizations WT1/KO1, KO1/WT2, WT2/KO2 and KO2/WT1. MA plot and heatmap from hippocampus (A) and cortex (B). MA plots plot the average intensities versus the log_2_ ratios. The dots are colored green and red if they are classified as down- and up-regulated, respectively. Data are based on the corrected p-values in combination with the fold change. Heatmaps are shown for the differentially expressed genes between both genotypes. Gene symbols are indicated at the right.
(TIF)

Table S1 **Primer sequences for regular and quantitative PCR.**
(DOC)
