# Supplementary figures and images for: Generation and Characterization of an Nxf7 Knockout Mouse to Study NXF5 Deficiency in a Patient with Intellectual Disability
Source: PLoS One. 2013 May 13;8(5):e64144. doi: 10.1371/journal.pone.0064144 (PMC3652825; doi:10.1371/journal.pone.0064144)

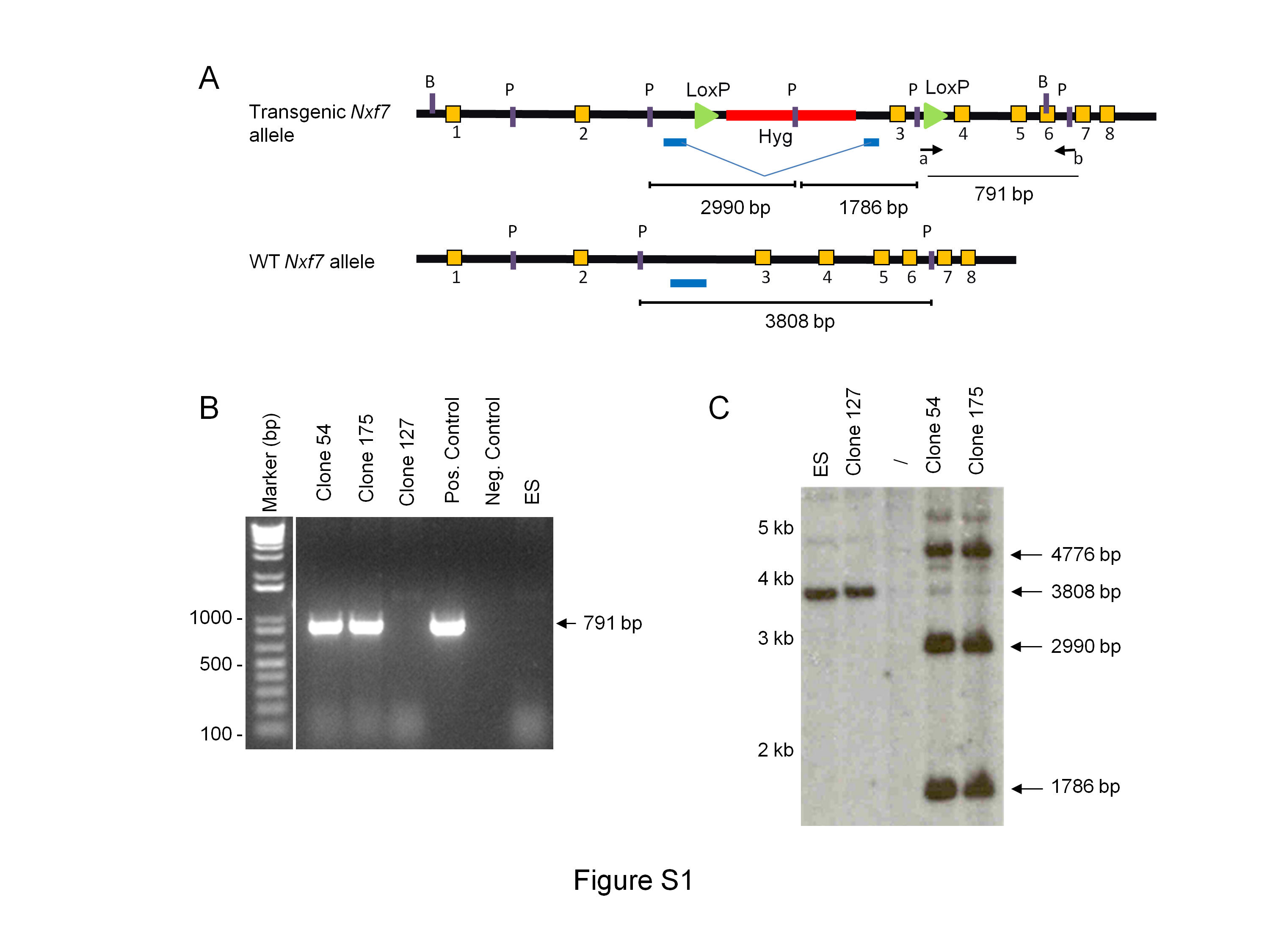

Supplement: Figure S1 — Confirmation of correctly targeted recombination in clones 54 and 175. (A) schematic representation of the transgenic Nxf7-targeted allele and the WT allele with the localization of the probes used in Southern blot. B, BamHI; P, PstI site; Hyg, hygromycine-resistant marker. Position of the probe in Nxf7 intron 2 is given as a horizontal blue bar; positions of primers a and b as black arrows. (B) PCR on genomic DNA of transfected ES clones with primer a, binding in the loxP sequence, and primer b annealing in exon 6 just outside the transfected construct. The targeting plasmid used for transfection was the positive control. This plasmid harbours the 8.7 kb BamHI (indicated by ‘B’) fragment shown on the construct of the ‘Transgenic Nxf7 allele’. (C) Southern blot analysis on PstI-digested DNA of embryonic stem (ES) WT cells and ES clones 54, 127 and 175 with the 32P-labeled probe. (TIF) [file pone.0064144.s001.tif]

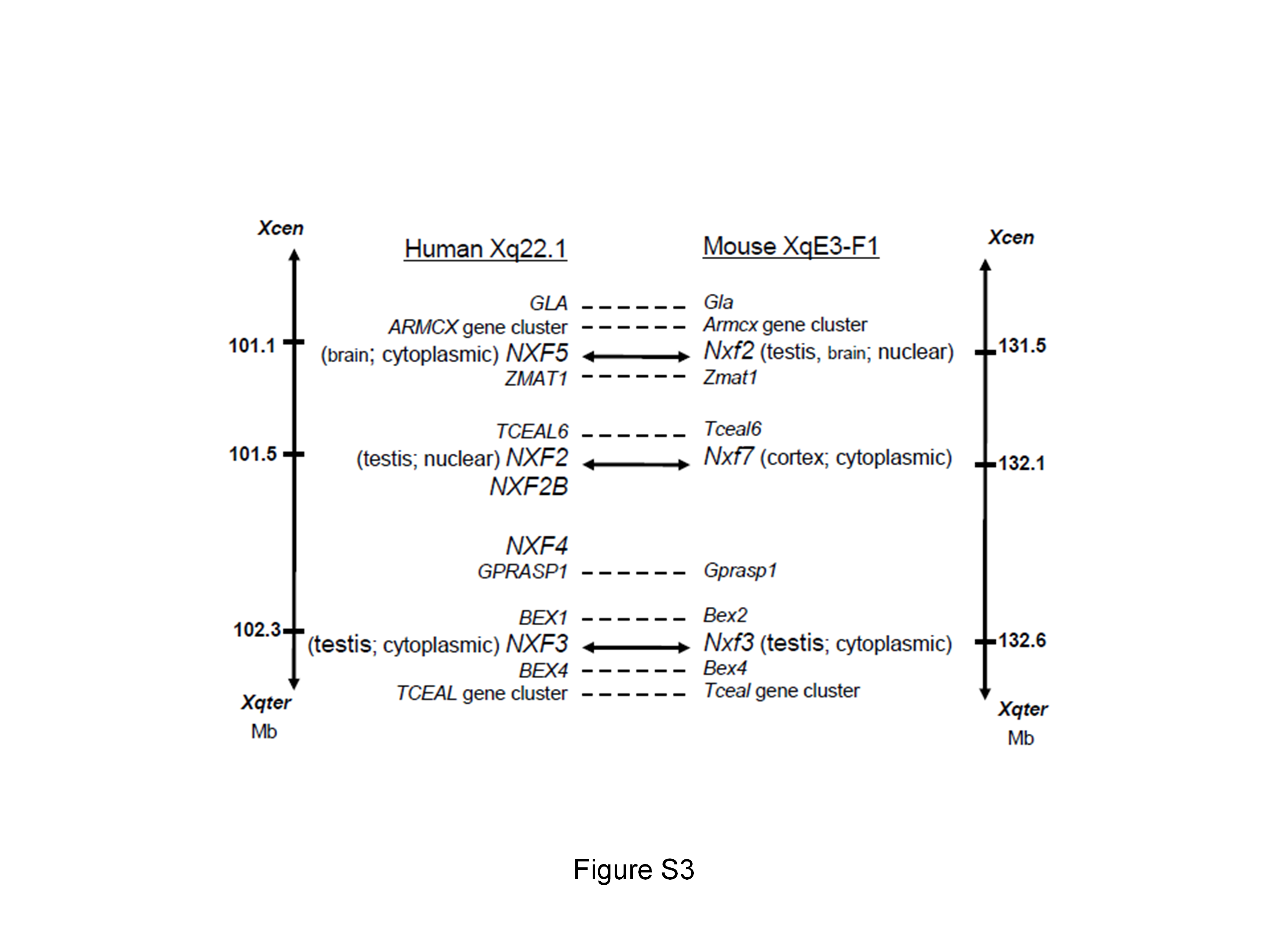

Supplement: Figure S3 — Schematic view of the syntenic regions in man (Xq22.1) and mouse (XqE3-F1). The orthology of genes within this highly conserved region is illustrated. According to the immediate flanking and neighbouring genes (GLA, ARMCX, ZMAT1, TCEAL6, GPRASP1, BEX1, BEX4), mouse Nxf2 is syntenic with human NXF5, and mouse Nxf7 with human NXF2. However, based on highest expression and subcellular localization data (indicated in between brackets) as well as other functional characteristics mentioned in the manuscript, the functional equivalent of Nxf2 should be NXF2, and that of NXF5 should be Nxf7. Note that in human, two additional NXF genes are present: NXF2B and the pseudogene NXF4. Positions (in Mb) on the X chromosome are given at the left and right. (TIF) [file pone.0064144.s003.tif]

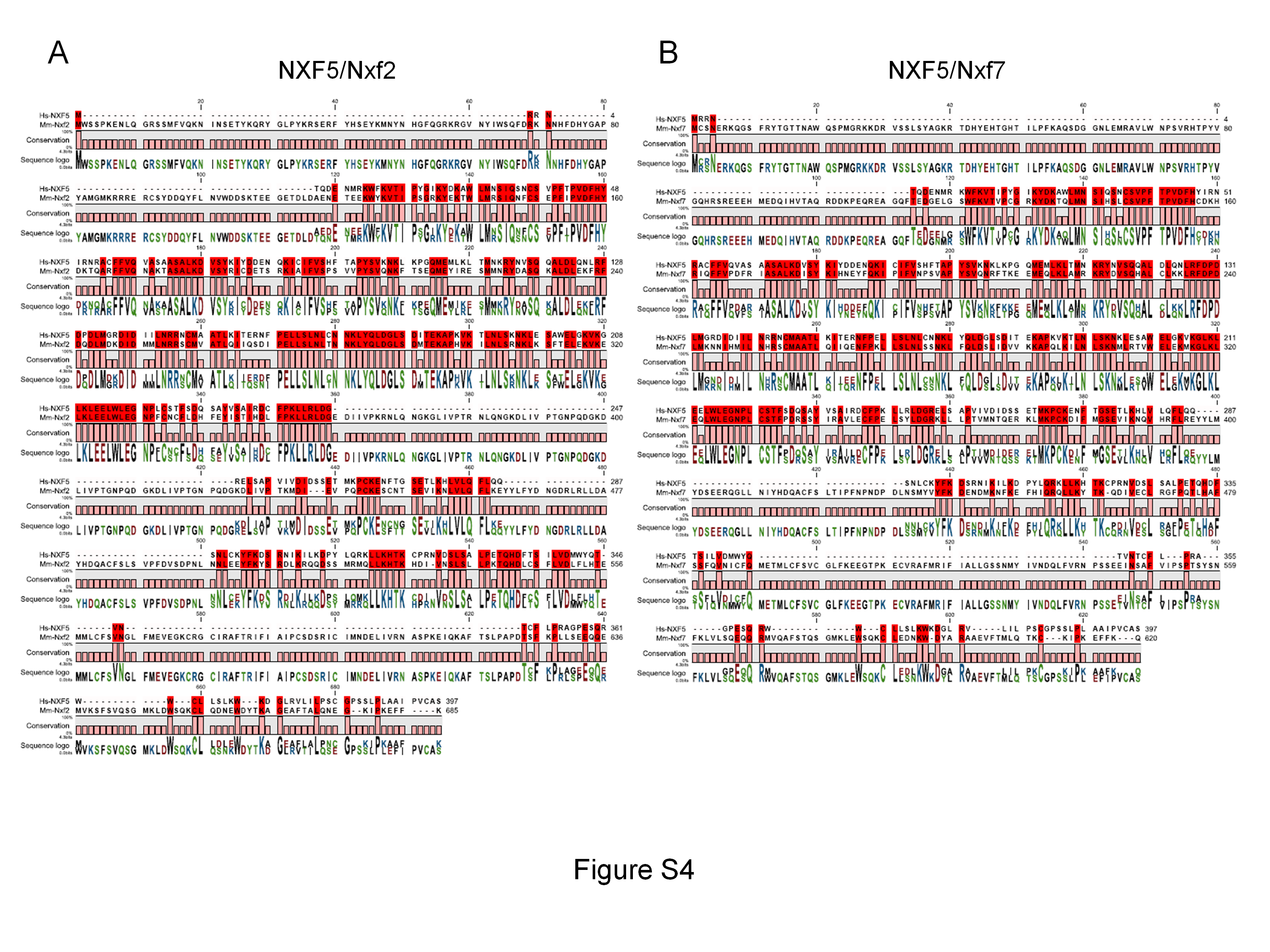

Supplement: Figure S4 — Protein alignment of NXF5 with Nxf2 or Nxf7. The human NXF5 protein sequence was aligned with that of mouse Nxf2 (A) or Nxf7 (B) using CLC DNA workbench software. Identical residues are highlighted in red. (TIF) [file pone.0064144.s004.tif]

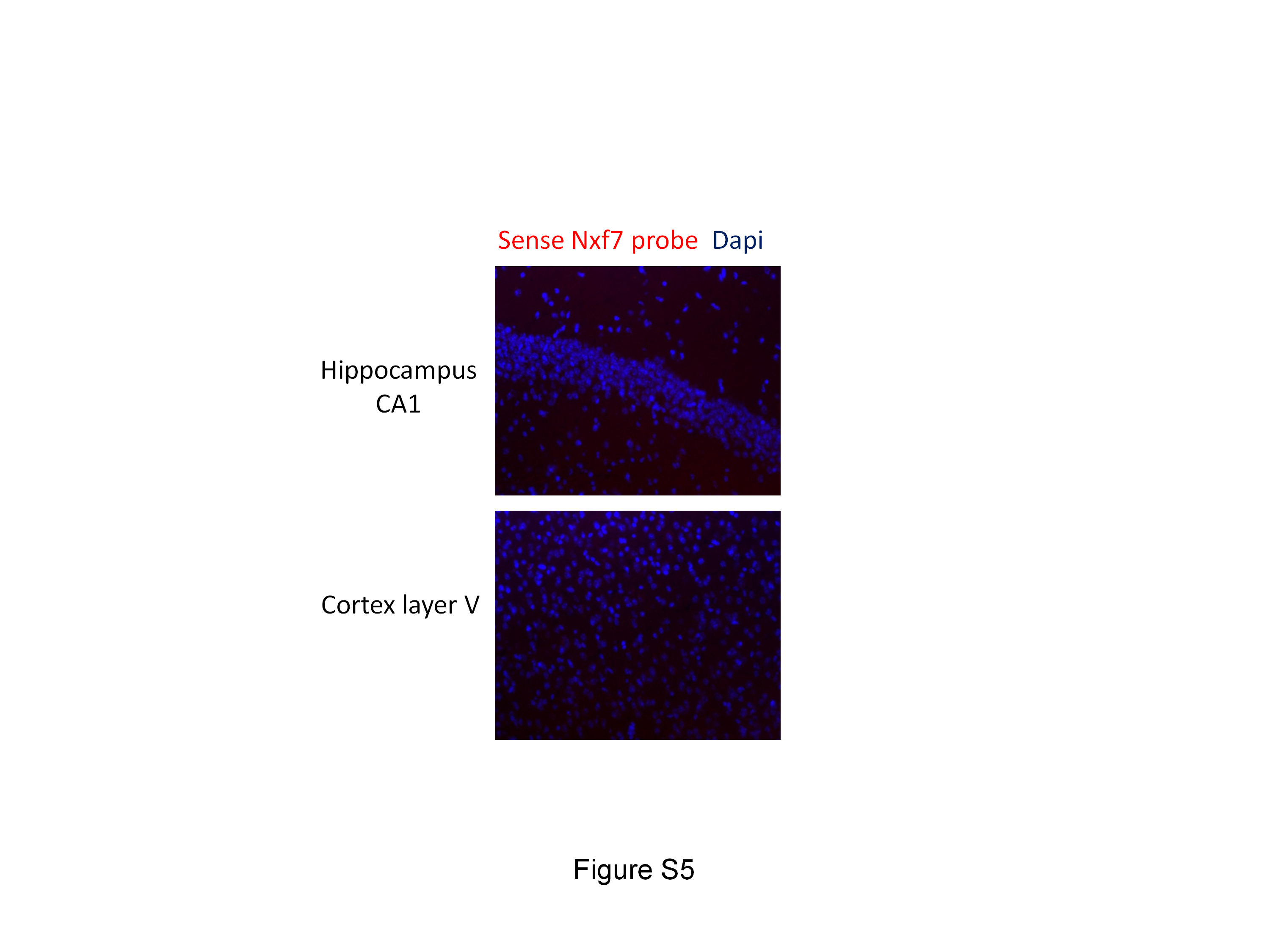

Supplement: Figure S5 — In situ hybridisation with the Nxf7 sense probe on brain slices revealed no specific staining. Representative pictures are shown for the CA1 region of the hippocampus and layer V of the cortex. The assay was done in duplicate. Scale bar, 100 µm. (TIF) [file pone.0064144.s005.tif]

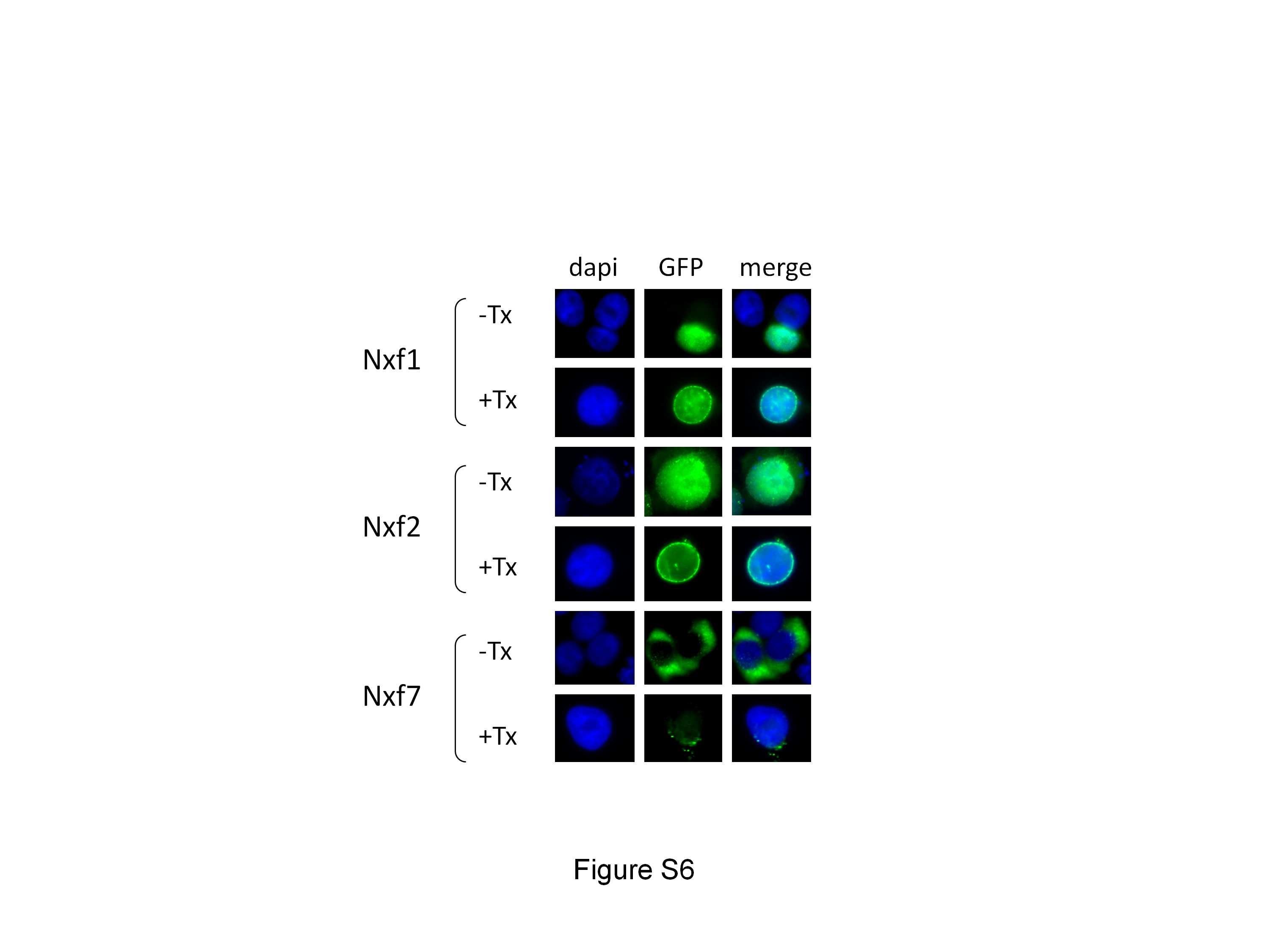

Supplement: Figure S6 — Subcellular localization of mouse Nxf proteins in HEK293T cells. The open reading frame of Nxf1, Nxf2 and Nxf7 were cloned in the pEGFP vector and 0.5 µg plasmid was transfected with the FuGENE6 transfection reagent. Cells were incubated for 20 hr and fixed with 4% formaldehyde for 15 min. Fluorescent signals were visualised with a MRC1024 confocal microscope. –Tx: without Triton-X100 treatment;+Tx; with Triton-X100 treatment. The magnifications used here slightly differ between images to accommodate the most optimal visualization. (TIF) [file pone.0064144.s006.tif]

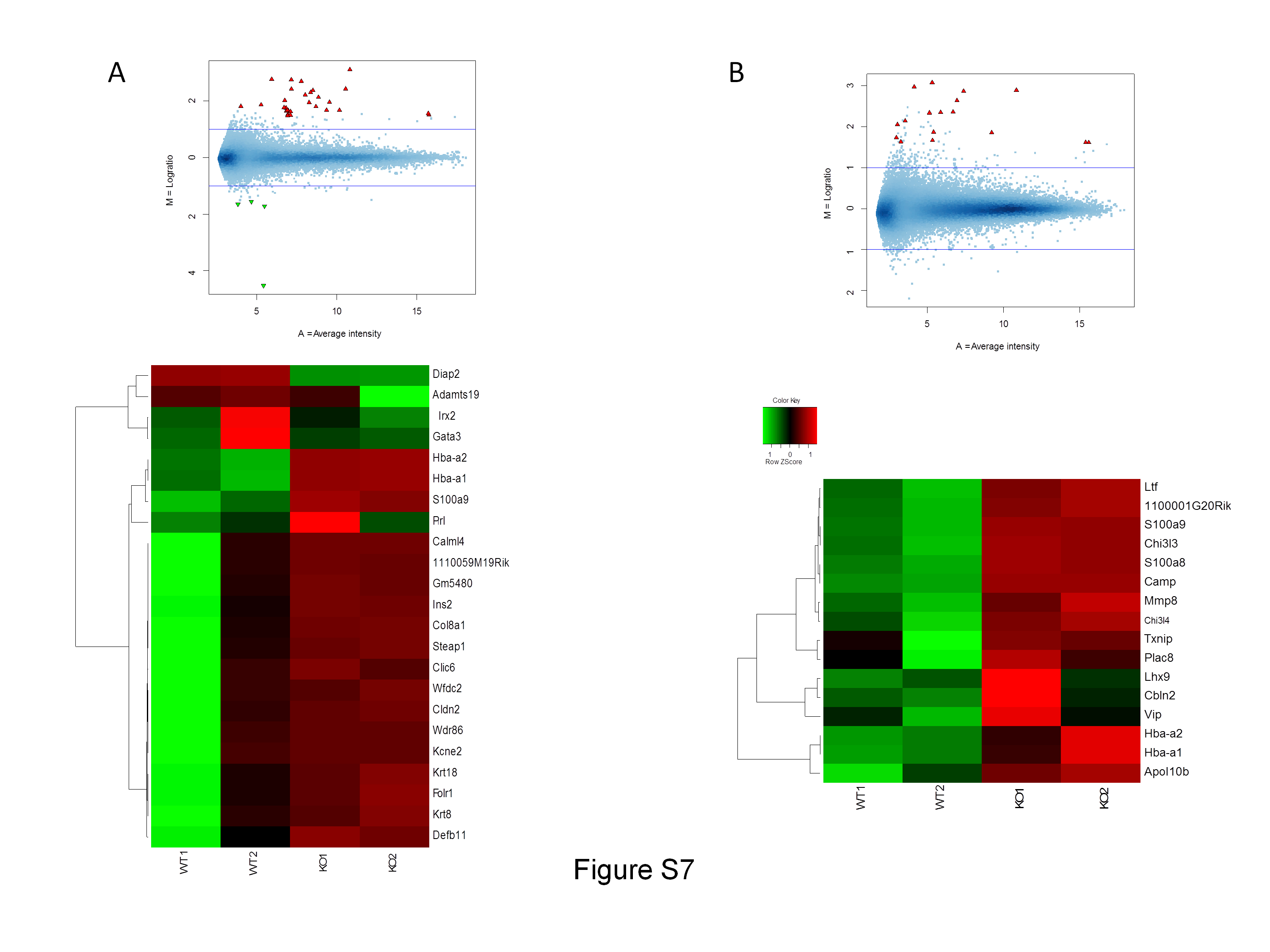

Supplement: Figure S7 — Microarray expression analysis of hippocampal and cortical RNA samples from male WT and Nxf7 KO littermates. Combined analysed data are shown for the hybridizations WT1/KO1, KO1/WT2, WT2/KO2 and KO2/WT1. MA plot and heatmap from hippocampus (A) and cortex (B). MA plots plot the average intensities versus the log2 ratios. The dots are colored green and red if they are classified as down- and up-regulated, respectively. Data are based on the corrected p-values in combination with the fold change. Heatmaps are shown for the differentially expressed genes between both genotypes. Gene symbols are indicated at the right. (TIF) [file pone.0064144.s007.tif]
