# Supplementary material for: Generation and Characterization of an Nxf7 Knockout Mouse to Study NXF5 Deficiency in a Patient with Intellectual Disability
Source: PLoS One. 2013 May 13;8(5):e64144. doi: 10.1371/journal.pone.0064144 (PMC3652825; doi:10.1371/journal.pone.0064144)
Supplement: Table S1 — Primer sequences for regular and quantitative PCR. (DOC) [file pone.0064144.s008.doc]

| **Table S1. Primer sequences for regular and quantitative PCR** | | | | | |  | |  |  | | |  | |  |  | |
| --- | --- | --- | --- | --- | --- | --- | --- | --- | --- | --- | --- | --- | --- | --- | --- | --- |
|  |  |  |  |  |  | |  | | |  |  | |  | | |  |
| **For genotyping of mice by regular PCR** | |  |  |  | Male | | | | |  | Female | | | | |  |
| For primer | forward primer (5' to 3') | Rev primer | reverse primer (5' to 3') |  | WT | | KO | | |  | WT | | HTZ | | |  |
| loxP-f | GCGGCCATGGATCTGTTG | ex6-r | CAGAGGCAATGCGAAAGTCTG |  | no | | 800 bp | | |  | no | | 800 bp | | |  |
| ex3-f | ACCGATCATTATGAGCATACTGG | ex4-r | TCTTCCTCCCTACTCCTATGTTGC |  | 380 bp | | no | | |  | 380 bp | | 380 bp | | |  |
| int2-f | GCTACTGCCTTAGTCTCCACTGTC | ex4-r | TCTTCCTCCCTACTCCTATGTTGC |  | 920 bp | | 323 bp | | |  | 920 bp | | 323/920 bp | | |  |
|  |  |  |  |  |  | |  | | |  |  | |  | | |  |
|  |  |  |  |  |  | |  | | |  |  | |  | | |  |
| **For expression analysis of mouse *Nxf* genes by regular PCR** | | |  |  |  | |  | | |  |  | |  | | |  |
| For primer | forward primer (5' to 3') | Rev primer | reverse primer (5' to 3') |  | size | |  | | |  |  | |  | | |  |
| Nxf1-f | CGAGTTTCACTATGAAAATACACG | Nxf1-r | ATGTTCTGGGCCACCAAATC |  | 279 bp | |  | | |  |  | |  | | |  |
| Nxf2-f | AATGAAACTGAGGAGAAATGGT | Nxf2-r | CGGGAGAGGTTCAGGATCTTA |  | 563 bp | |  | | |  |  | |  | | |  |
| Nxf3-f | CAATCCCAACATTGCCTTTATGC | Nxf3-r | CTGCCGATCTCCATTGTCATAG |  | 595 bp | |  | | |  |  | |  | | |  |
| Nxf7-f | CATGCTCTCTGCCTCAAAAAG | Nxf7-r | TCTCCAACTCCCACACTGTTC |  | 259 bp | |  | | |  |  | |  | | |  |
| Actb-f | GAGAGGTATCCTGACCCTGAAGT | Actb-r | ATCTTCATGAGGTAGTCCGTCAG |  | 393 bp | |  | | |  |  | |  | | |  |
|  |  |  |  |  |  | |  | | |  |  | |  | | |  |
| **For analysis of mouse *Nxf7* mRNA abundance by regular PCR** | | |  |  | Male | | | | |  | Female | | | | |  |
| For primer | forward primer (5' to 3') | Rev primer | reverse primer (5' to 3') |  | WT | | KO | | |  | WT | | HTZ | | |  |
| ex1-f | AGGCCAAAAGACAAGAAAGATGTGC | ex5-r | CTGCAAAGGCTGTGGATTGAAT |  | 459 bp | | 304 bp | | |  | 459 bp | | 459/304 bp | | |  |
| ex3-f | ACCGATCATTATGAGCATACTGG | ex5-r | CTGCAAAGGCTGTGGATTGAAT |  | 320 bp | | no | | |  | 320 bp | | 320 bp | | |  |
| ex8-f | CATGCTCTCTGCCTCAAAAAG | ex16-r | GGAGAAGCAGGCCTGGTCA |  | 579 bp | | 579 bp | | |  | 579 bp | | 579 bp | | |  |
| Actb-f | GAGAGGTATCCTGACCCTGAAGT | Actb-r | ATCTTCATGAGGTAGTCCGTCAG |  | 393 bp | | 393 bp | | |  | 393 bp | | 393 bp | | |  |
|  |  |  |  |  |  | |  | | |  |  | |  | | |  |
|  |  |  |  |  |  | |  | | |  |  | |  | | |  |
| **For expression analysis of mouse *Nxf2 and Nxf7* by qPCR** | | |  |  |  | |  | | |  |  | |  | | |  |
| For primer | forward primer (5' to 3') | Rev primer | reverse primer (5' to 3') |  |  | |  | | |  |  | |  | | |  |
| Nxf2ex21-22-f | CTCTGTGCAGTCTGGAATGAAA | Nxf2ex21-22-r | TTGGTGTAGTCCCACTCG |  |  | |  | | |  |  | |  | | |  |
| Nxf7ex3-4-f | GAGTGATGGTGGAAATCTGGAG | Nxf7ex3-4-r | CCAACATAAGGTGTGTGTCTTACTGA |  |  | |  | | |  |  | |  | | |  |
| Nxf7ex21-22-f | CTTTCTCCACCCAGTCGG | Nxf7ex21-22-r | CTTCAGCAGCTCTGGCAT |  |  | |  | | |  |  | |  | | |  |
| Actb-f | ACCCACACTGTGCCCATCTAC | Actb-r | AGCCAAGTCCAGACGCAGG |  |  | |  | | |  |  | |  | | |  |
| Gusb-f | CACTAATGTGGAGCAAGACATC | Gusb-r | GCACTTCTAGCTGGAAATGTT |  |  | |  | | |  |  | |  | | |  |
| Hprt-f | CATTATGCCGAGGATTTGG | Hprt-r | GCAAGTCTTTCAGTCCTGT |  |  | |  | | |  |  | |  | | |  |
|  |  |  |  |  |  | |  | | |  |  | |  | | |  |
|  |  |  |  |  |  | |  | | |  |  | |  | | |  |
|  |  |  |  |  |  | |  | | |  |  | |  | | |  |
| **For expression analysis of human *NXF2* and *NXF5* by qPCR** | | |  |  |  | |  | | |  |  | |  | | |  |
| For primer | forward primer (5' to 3') | Rev primer | reverse primer (5' to 3') |  |  | |  | | |  |  | |  | | |  |
| NXF2-TMf | CCGGTTGATTTCCACTACGTCC | NXF2-TMr | TGCGGAGGCAGCGCTAGCA |  |  | |  | | |  |  | |  | | |  |
| NXF5-TMf | GCACACAAAATGTCCACGAAA | NXF5-TMr | ACCAGGATGGAGGTGAAGTCA |  |  | |  | | |  |  | |  | | |  |
| GUSB-TMf | AGAGTGGTGCTGAGGATTGG | GUSB-TMr | CCCTCATGCTCTAGCGTGTC |  |  | |  | | |  |  | |  | | |  |
| HPRT-TMf | TGACACTGGCAAAACAATGCA | HPRT-TMf | GGTCCTTTTCACCAGCAAGCT |  |  | |  | | |  |  | |  | | |  |
